# Monocarboxylate Transporter 13 (MCT13/SLC16A13) Functions as a Novel Plasma Membrane Oligopeptide Transporter

**DOI:** 10.3390/nu15163527

**Published:** 2023-08-10

**Authors:** Kei Higuchi, Misato Kunieda, Koki Sugiyama, Ryuto Tomabechi, Hisanao Kishimoto, Katsuhisa Inoue

**Affiliations:** 1 Department of Biopharmaceutics, School of Pharmacy, Tokyo University of Pharmacy and Life Sciences, 1432-1 Horinouchi, Tokyo 192-0392, Japan; higuchi@toyaku.ac.jp (K.H.); y174054@toyaku.ac.jp (M.K.); y141106@toyaku.ac.jp (K.S.); tomabechi.ryuto@kitasato-u.ac.jp (R.T.); kisimoto@toyaku.ac.jp (H.K.); 2 Laboratory of Pharmaceutics, Kitasato University School of Pharmacy, 5-9-1 Shirokane, Tokyo 108-8641, Japan

**Keywords:** monocarboxylate transporter 13, oligopeptide, cephradine, membrane transport, intestinal epithelium, membrane potential, transporter, protein–protein interaction, CD147

## Abstract

*SLC16A13*, which encodes the monocarboxylate transporter 13 (MCT13), is a susceptibility gene for type 2 diabetes and is expressed in the liver and duodenum. Some peptidase-resistant oligopeptides are absorbed in the gastrointestinal tract and affect glycemic control in the body. Their efficient absorption is mediated by oligopeptide transporter(s) at the apical and basolateral membranes of the intestinal epithelia; however, the molecules responsible for basolateral oligopeptide transport have not been identified. In this study, we examined whether MCT13 functions as a novel basolateral oligopeptide transporter. We evaluated the uptake of oligopeptides and peptidomimetics in MCT13-transfected cells. The uptake of cephradine, a probe for peptide transport system(s), significantly increased in MCT13-transfected cells, and this increase was sensitive to membrane potential. The cellular accumulation of bioactive peptides, such as anserine and carnosine, was decreased by MCT13, indicating MCT13-mediated efflux transport activity. In polarized Caco-2 cells, MCT13 was localized at the basolateral membrane. MCT13 induction enhanced cephradine transport in an apical-to-basal direction across Caco-2 cells. These results indicate that MCT13 functions as a novel efflux transporter of oligopeptides and peptidomimetics, driven by electrochemical gradients across the plasma membrane, and it may be involved in the transport of these compounds across the intestinal epithelia.

## 1. Introduction

Intestinal epithelial transporters mediate the absorption of oligopeptides and peptidomimetics. Di-/tri-peptides and peptide-like drugs, such as β-lactam antibiotics, permeate the apical membrane of intestinal epithelia through H^+^-coupled oligopeptide transporter 1 (PEPT1) [1,2]. These compounds are metabolized in cells or released intact into the bloodstream through the basolateral membrane. Although the latter process involves transporter-mediated permeation at the basolateral membrane of the intestinal epithelial cells [3,4,5,6], the molecular entities of basolateral oligopeptide transporters remain unknown.

Several dietary peptides are to be absorbed intact from the gastrointestinal tract to improve glycemic control in humans and animals [7,8,9]. These bioactive peptides exhibit inhibitory effects on dipeptidyl peptidase IV (DPP4), which is involved in glucagon-like peptide-1 (GLP-1) inactivation, and therefore play an important role in glycemic regulation [10]. Therefore, bioactive peptides that escape from intracellular metabolism in enterocytes are transferred into the blood to suppress DPP4 activity. The bioactive peptides carnosine and anserine, which are naturally occurring dipeptides in animal foods, are resistant to gastrointestinal peptidases and can permeate through intestinal epithelial cells without hydrolysis [11]. The intestinal absorptive system(s) for carnosine and anserine are shared by other oligopeptides and peptidomimetics [12,13], in which apical PEPT1 and basolateral unknown oligopeptide transporter(s) are involved in the influx and efflux of peptides, respectively.

The solute transporter family 16 (SLC16) consists of 14 members, which are implicated in the influx and efflux transport of various endogenous metabolites. SLC16A1 was first identified as a prototypical monocarboxylate transporter (MCT1); therefore, the members of the family are annotated as MCTs. Although MCT1–4 transport metabolically important short-chain fatty acids (SCFAs) through the plasma membrane in an H^+^-coupling manner [14,15,16], other MCTs, such as MCT7, MCT9, and MCT12, mediate the transport of zwitterionic metabolites such as taurine, carnitine, and creatine, respectively [17,18,19]. MCT7 and MCT12 are characterized as facilitated diffusion-type transporters and function as influx or efflux transporters, depending on the substrate concentration gradient [17,19]. There are several orphan transporters in the SLC16 family, which have pathophysiological roles [20].

MCTs are known to be complexed with ancillary proteins, such as basigin/CD147 and/or embigin/GP70, at the plasma membrane. CD147 preferentially interacts with MCT1, MCT3, MCT4, and MCT12, whereas GP70 interacts with MCT2 and MCT7 as well as with MCT1 in the absence of CD147 [20,21]. Furthermore, these complexes affect the stable membrane expression of MCTs and regulate transport function [22]. Although there are controversial reports of the localizations of MCTs, the MCT and ancillary protein complexes are primarily localized to the basolateral membrane of intestinal epithelia and/or Caco-2 human intestinal epithelial cells [19,23,24,25].

*SLC16A13* encodes MCT13, which is a susceptibility gene for type 2 diabetes in Japanese and Chinese populations [26,27]. MCT13/SLC16A13 is primarily expressed in the liver, but also in the duodenum, kidney, and various epithelial cell lines [24]. A recent study using Mct13-deficient mice revealed that Mct13 transports lactate and affects energy metabolism in the liver [28]; however, the substrate of human MCT13 remains unclear in the intestine. We hypothesized that MCT13 may function as a basolateral oligopeptide transporter in intestinal epithelial cells, considering the association of bioactive peptide transporters into systemic circulation with diabetes. In the present study, we show the MCT13-mediated transport of oligopeptides and peptidomimetics in MCT13/CD147 double-transfected cells. We also demonstrate that MCT13 transports the peptidomimetic cephradine as a probe substrate of the intestinal peptide transport system(s) in a membrane potential-sensitive manner. Furthermore, the permeation of cephradine is clearly enhanced by MCT13 expression at the basolateral membrane of Caco-2 cells. MCT13 caused a decrease in the intracellular accumulation of oligopeptides, such as carnosine and anserine, in the steady state.

## 2. Materials and Methods

### 2.1. Materials and Reagents

All of the reagents used were of reagent grade available from commercial sources. Cell culture medium and fetal bovine serum (FBS) were purchased from Fujifilm Wako Pure Chemical (Osaka, Japan) and Nichirei Biosciences Inc. (Tokyo, Japan), respectively. We obtained pMK243 (Tet-OsTIR1-PURO) and AAVS1-T2 CRIPR/pX330 from Addgene as gifts from Masato Kanemaki (Addgene plasmid #72835; RRID: Addgene_72835, and #72833; RRID: Addgene_72833) [29]. pBiFC-VN155 (I152L) and pBiFC-VC155 were gifts from Chang-Deng Hu (Addgene plasmids #27097; RRID: Addgene_27097, and #22011; RRID: Addgene_22011) [30,31].

### 2.2. Construction of Expression Vectors

Human *SLC16A13* (NM_201566.3), *CD147* (NM_001728.4), and *GP70* (NM_198449.3) were cloned into pEGFP-C1 (Clontech, CA, USA), pcDNA 3.1 Hygro (+) (Thermo Fisher Scientific, Waltham, MA, USA), pCI-Neo (Promega, Madison, WI, USA), pBiFC-VN155 (I152L), or pBiFC-VC155. To prepare Caco-2 cells with tetracycline inducible expression of MCT13/SLC16A13, the cDNA of EGFP-tagged SLC16A13 was inserted into pMK243 vectors. The sequences of all constructs were examined by the Eurofins Genomics DNA sequencing service (Tokyo, Japan) or a DNA sequencer (ABI Prism 3100 Genetic Analyzer, Applied Biosystems, Foster City, CA, USA).

### 2.3. Cell Lines and Culture Conditions

HEK293T cells and Caco-2 cells were obtained from the American Type Culture Collection (ATCC) (Cat. No. CRL-3216 and HTB-37, respectively). These cells were cultured in Dulbecco’s Eagle’s Minimum Essential Medium, supplemented with fetal bovine serum at a final concentration of 10% and antibiotics (penicillin, 100 mU/mL; streptomycin, 100 μg/mL) (Thermo Fisher Scientific) with or without 1% nonessential amino acids (Thermo Fisher Scientific).

### 2.4. Preparation of Caco-2 Cells Conditionally Expressing EGFP-Tagged MCT13

Caco-2 cells conditionally expressing MCT13 under the control of Tet-on promoters (Caco-2-Tet-MCT13) were prepared according to the previous report [19]. Briefly, Caco-2 cells were transfected with the plasmids of EGFP-tagged MCT13/pMK243 and AAVS1-T2 CRIPR/pX330 for editing the genome of Caco-2 at the adeno-associated virus integration site (AAVS) safe harbor site. The transfected cells were then selected by a culture medium containing puromycin dihydrochloride (12.5 μg/mL). The expression and localization analysis of EGFP-tagged MCT13 in Caco-2-Tet-MCT13 cells were performed by fluorescence microscopy (BZ-X810; Keyence, Osaka, Japan). Caco-2-Tet-MCT13 cells were used for localization and functional analysis at the passage number range of 15 to 22 after the puromycin selection. The tight junction integrity of Caco-2-Tet-MCT13 cells was confirmed by high TEER values (over 900 Ω·cm^2^) and by the low permeation of lucifer yellow (less than 1% of the total amount). In addition, we checked the function of PEPT1 in Caco-2-Tet-MCT13, which was comparable with that in the original Caco-2 cells.

### 2.5. Localization Analysis

HEK293T cells were seeded on poly-lysine-coated culture cover glass (Matsunami Glass, Osaka, Japan) and transfected with plasmids of MCT13 and/or CD147 or GP70 by using polyethyleneimine. After 48 h of incubation, the cells were stained by PlasMem Bright Red (Dojindo laboratories, Kumamoto, Japan) and observed by fluorescence microscopy (BZ-X810). Caco-2-Tet-MCT13 cells were cultured on a Falcon cell culture insert membrane for 24 days and treated with 5 μg/mL doxycycline and 5 mM sodium butyrate for 2 days. The cells were fixed by 4% paraformaldehyde at room temperature for 10 min. The cell culture insert was mounted by using a mounting medium with DAPI (Vector Laboratories, Burlingame, CA, USA) and observed by fluorescence microscopy (BZ-X810).

### 2.6. Uptake Study

HEK293T cells were seeded on poly-lysine-coated 24-well or 48-well plates. The cells were transfected with plasmids of MCT13 and/or CD147. The cells transfected with the plasmid of CD147 were used as mock-transfected cells. For co-expression of MCT13 and PEPT2, HEK293T cells were transfected with plasmids of MCT13 and CD147 and cultured for 24 h, and then further transfected with plasmids of PEPT2. Caco-2-Tet-MCT13 cells were seeded on 24-well plates and treated with 5 μg/mL doxycycline and 5 mM sodium butyrate. After 48 h transfection or treatment, uptake experiments were performed using HBBS buffer or HBSS-modified buffer for removing Na^+^ and/or Cl^−^, in which NaCl was replaced with mannitol, KCl, Na-gluconate, or K-gluconate. To deplete membrane potential in cells, pretreatment of Ba^2+^ and co-incubation of trifluoromethoxy carbonylcyanide phenylhydrazone (FCCP) were used. Pretreatment of Ba^2+^ at 2 mM was performed for 30 min in the modified HBSS buffer without SO_4_^2−^ and CO_3_^2−^. FCCP at 10 μM was co-incubated with cells at the same time as cephradine uptake. After uptake experiments, the intracellular contents of Gly-Sar, cephradine, carnosine, and anserine were measured by a liquid chromatography–tandem mass spectrometer (Waters Acquity UPLC H-Class equipped with the Xevo TQD system; Milford, MA, USA) under the condition as shown in Table S1. Cells were suspended in distilled water, and the cell suspensions were deproteinated by adding acetonitrile. The samples were passed through a 0.45-μm PVDF filter (FastRemover for Protein; GL Sciences, Tokyo, Japan), and the filtrates were used for analysis. Analytes were separated by the LC system at a 0.6 mL/min flow rate on an Intrada Amino acid analytical column (50 × 3 mm, 3 μm) (Imtakt, Kyoto, Japan), which can separate dipeptide and peptide derivatives including anserine and carnosine, especially [32]. The separation was achieved by using a gradient program composed of solvent A (ammonium formate buffer (20 mM)) and solvent B (acetonitrile). The separation was performed by a column set at 35 °C. Mass Lynx software version 4.1 software was used to control the instrument and to collect data. Protein amounts in the cell suspensions were measured by the Pierce BCA protein assay kit (Thermo Fisher Scientific). Cellular uptake amount was shown as the cell-to-medium ratio by dividing the intracellular contents by the test compound concentration in the uptake buffer. The uptake amount was normalized to the cellular protein content.

### 2.7. Directional Transport Study

A directional transport study was performed by using Caco-2-Tet-MCT13 cells cultured on Falcon cell culture inserts (3 μm pores, 353090, BD-Falcon; Franklin Lakes, NJ, USA). The cells were cultured in a normal culture medium for 24–27 days and then treated with 5 μg/mL doxycycline and 5 mM sodium butyrate for 2 days to induce MCT13 expression. HBSS-MES buffer (pH 6.0) and HBSS-HEPES buffer (pH 7.4) were added to the upper chamber and lower chamber, respectively. The permeation values of cephradine and lucifer yellow across the Caco-2-Tet-MCT13 cells for apical-to-basal (A-to-B) and basal-to-apical (B-to-A) directions were evaluated. HBSS-MES buffer (pH 6.0) including cephradine (500 μM) and lucifer yellow (500 μM), was added to the upper chamber or lower chamber to initiate the transport experiment. Samples were taken from the other side chamber at the designed time and stored at −20 °C before determining the contents of cephradine and lucifer yellow. The contents were quantified by LC-MS/MS or a fluorescent microplate reader (Varioskan Flash 2.4; Thermo Fisher Scientific). Transport amounts were calculated by dividing the sample contents by the initial concentration in the transport buffer.

### 2.8. Accumulation Study

HEK293T cells were seeded on 24-well plates coated with poly-L-lysine. The cells were transfected with plasmids of MCT13 and CD147, as described above. After 24 h of transfection, the cell culture medium was supplemented with 500 μM of the indicated oligopeptide. After 24 h incubation, the accumulated oligopeptides were measured by LC-MS/MS.

### 2.9. Statistics

All measurements were always performed in more than triplicate, and the experiments were repeated twice with separate cultures. Data are always shown as means ± S.D. Statistical analyses and graphing were performed in GraphPad Prism 9.4 software. Statistical differences between the control and experimental groups were judged by unpaired *t*-tests, or by one-way analysis or two-way analysis of variance, followed by Dunnett’s or Sidak’s test for multiple comparisons; *p* < 0.05 was considered significant. For these statistical tests, the normality was confirmed using the GraphPad Prism 9.4 software.

## 3. Results

### 3.1. Enhanced Expression of MCT13 at the Plasma Membrane by CD147

**Figure 1:**
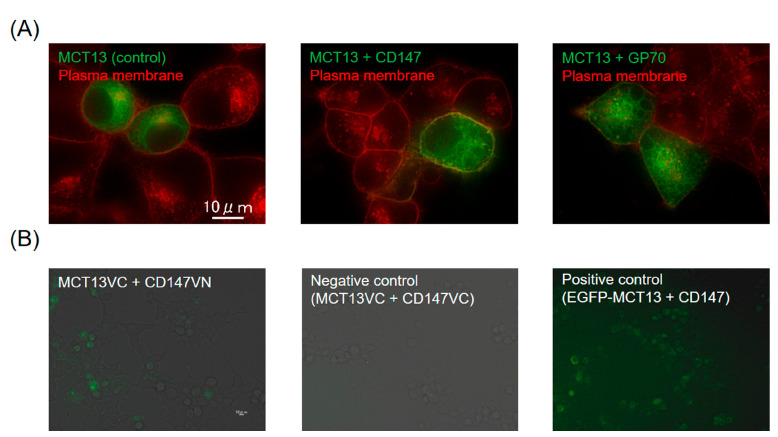
Ancillary protein CD147 enhances the plasma membrane localization of MCT13. (**A**) Effect of CD147 and GP70 on the intracellular localization of MCT13 in HEK239T cells. HEK293T cells were transfected with plasmids of EGFP-tagged MCT13 (green) and CD147 or GP70. The plasma membrane of cells was stained by PlasMem Bright Red (red). (**B**) Bi-FC assay for interactions between MCT13 and CD147. VN and VC mean the *N-* and *C*-terminal fragments of Venus fluorescent protein, respectively. HE293T cells were transfected with MCT13-VC and CD147-VN or -VC. EGFP-tagged MCT13 was used as a positive control. Venus fluorescent signals were detected by fluorescence microscopy. The obtained fluorescent images were merged with phase contrast images.

To examine whether MCT13 is localized at the plasma membrane of HEK293T cells, we analyzed the subcellular localization of EGFP-tagged MCT13 in the presence or absence of the ancillary proteins, CD147 and GP70 (Figure 1A and Figure S1). MCT13 was partially localized at the plasma membrane of HEK293T cells co-expressing MCT13 and CD147, although it was primarily localized in the intracellular compartments of cells expressing only MCT13. In contrast, localization was unchanged in HEK293T cells co-expressing MCT13 and GP70. Furthermore, the BiFC assay detected Venus fluorescent signals in HEK293T cells co-expressing the *C*-terminal Venus-fragment fused MCT13 (MCT13-VC) with *N*-terminal Venus-fragment fused CD147 (CD147-VN), but not in cells co-expressing MCT13-VC with *C*-terminal Venus-fragment fused CD147 (CD147-VC) (Figure 1B). The results suggest that the interaction between MCT13 with CD147 enhances the plasma membrane expression of MCT13 in HEK293T cells. Therefore, HEK293T cells co-expressing MCT13 and CD147 were used for subsequent uptake studies.

### 3.2. MCT13-Mediated Transport of Oligopeptides and Peptidomimetics

**Figure 2:**
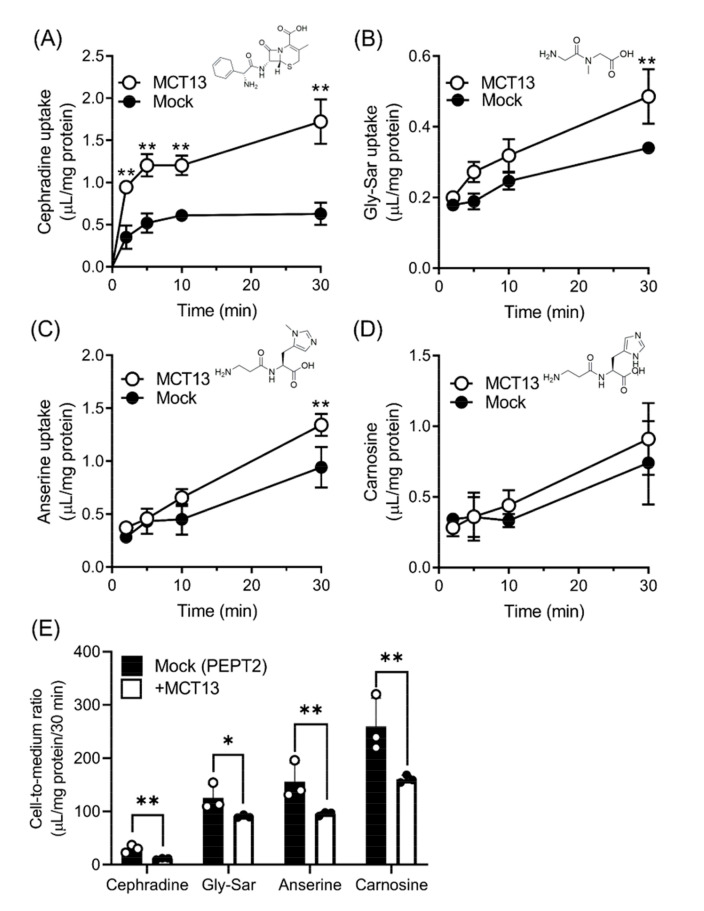
MCT13 mediates the uptake and efflux of oligopeptides and peptidomimetics. (**A**–**D**) Uptakes of cephradine (**A**), Gly-Sar (**B**), anserine (**C**), and carnosine (**D**) by HEK293T expressing EGFP-tagged MCT13 and CD147. Uptake of each substrate was measured at 500 μM in HBBS-modified (Na^+^-free) buffer (pH 7.4) for the indicated time. (**E**) Effect of MCT13 on PEPT2-mediated oligopeptide and peptidomimetics uptakes. HEK293T cells were transfected with the plasmid of CD147 with or without plasmids of EGFP-tagged MCT13. The cells were re-transfected with plasmids of PEPT2. Uptake of these compounds was measured at 20 μM in Na^+^-free buffer (pH 6.5) for 30 min. Each point represents the mean ± S.D. (*n* = 3). *, *p* < 0.05, and **, *p* < 0.01, compared to the corresponding mock-transfected cells by two-way ANOVA with Sidak’s multiple comparisons test (**A**–**D**) or by unpaired *t*-test (**E**).

To determine whether MCT13 transports oligopeptides and peptidomimetics, we measured the uptake of these compounds in HEK293T cells expressing EGFP-tagged MCT13 and CD147. Cephradine, glycylsarcosine (Gly-Sar), anserine, and carnosine were used as uptake probes, because these compounds are known substrates of the H^+^-coupled oligopeptide transporter (PEPT) and resistant to hydrolysis by peptidases [11]. Of these compounds, the uptake of cephradine in MCT13-transfected cells was clearly increased with time compared with that in mock-transfected cells (Figure 2A). In contrast, the uptake of Gly-Sar and anserine showed a marginal increase only at 30 min, whereas that of carnosine was unchanged in MCT13-transfected cells (Figure 2B–D). To determine whether a concentration gradient between the intracellular and extracellular environment affects the MCT13-mediated transport, we evaluated the uptake of oligopeptides and peptidomimetics using double MCT13/PEPT2-transfected cells (Figure 2E). PEPT2 mediates a concentrative uptake and causes an accumulation of substrates into cells. In addition, PEPT2 is known to be a high-affinity and low-capacity transporter and able to load substrates into cells at low substrate concentrations, whereas PEPT1 is a low-affinity and high-capacity transporter. As shown on the *Y*-axis of Figure 2E, the uptake of PEPT2 substrates (cephradine, Gly-Sar, anserine, and carnosine) in PEPT2-transfected cells was greater compared with that in MCT13- or mock-transfected cells (Figure 2A–D). In contrast, the uptake of cephradine, Gly-Sar, anserine, and carnosine in double MCT13/PEPT2-transfected cells was significantly decreased compared with that in single PEPT2-transfected cells. These results suggest that MCT13 may have the ability to mediate both the influx and efflux of oligopeptides and peptidomimetics. It is possible that MCT13 works as an efflux transporter due to its notable efflux activity in comparison to its influx activity. For subsequent studies, cephradine, an anionic peptide-like compound, was selected as a transport probe for MCT13 because the uptake was distinct among the tested compounds. Furthermore, we used the plasmid of untagged MCT13 for detailed characterization.

### 3.3. Characteristics of Cephradine Transport by MCT13

**Figure 3:**
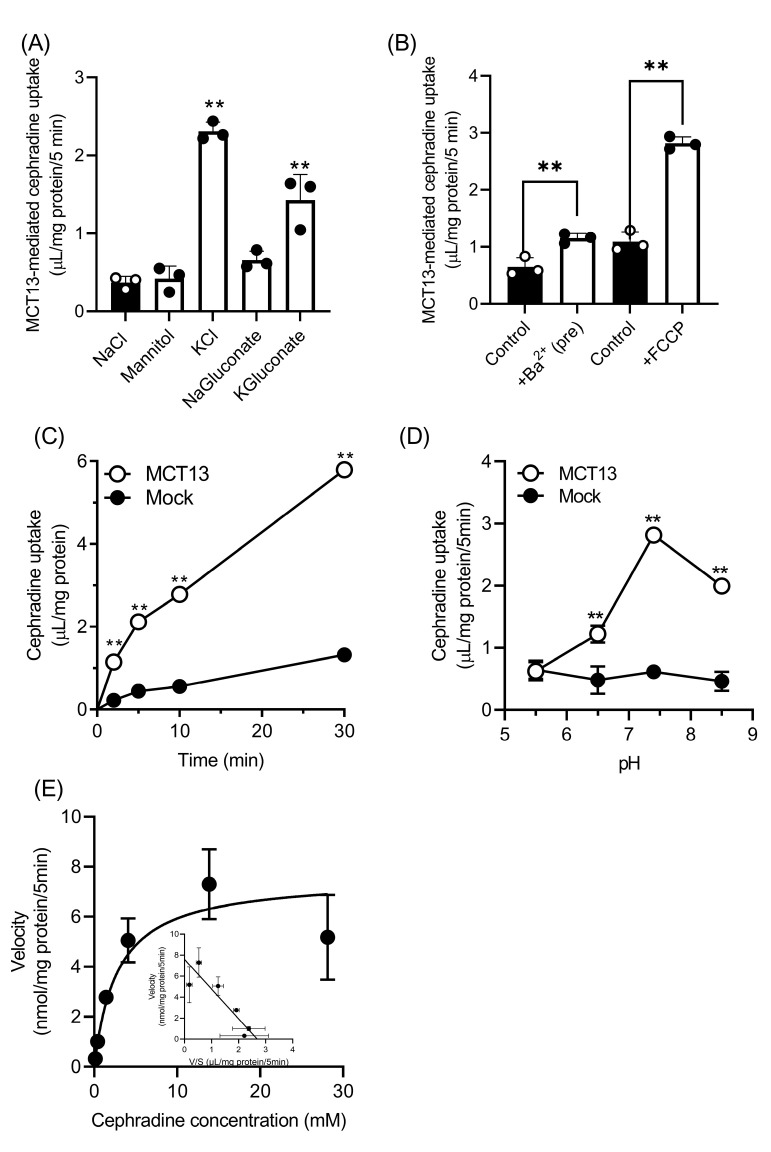
Depletion of membrane potential enhances MCT13-mediated cephradine uptake. Cephradine uptake was measured at 500 μM, except in (**E**), using HEK293T cells expressing MCT13 and CD147. (**A**) Effect of extracellular ions on MCT13-mediated cephradine uptake. Uptake in HBSS buffer (pH 7.4) or HBSS-modified buffer (pH 7.4) was measured. (**B**) Effect of Ba^2+^ pretreatment and FCCP co-incubation on MCT13-mediated cephradine uptake. For evaluation of the Ba^2+^ effect, the cells were preincubated with the modified HBSS buffer with or without 2 mM Ba^2+^. Cephradine uptake was measured in the modified HBSS buffer (pH 7.4) containing 2 mM Ba^2+^ or in HBSS buffer (pH 7.4) containing 10 μM FCCP. (**C**) Cephradine uptake was measured in KCl buffer (pH 7.4) for the designed time. (**D**) Effect of extracellular pH on MCT13-mediated cephradine uptake. The uptake was measured in KCl buffer at a pH of 5.5, 6.5, 7.4, and 8.5. (**E**) Concentration dependence of MCT13-mediated cephradine uptake. The uptake at 0.1 mM–30 mM was measured. Inset shows the Eadie–Hofstee plot. Each point represents the mean ± S.D. (*n* = 3). **, *p* < 0.01, compared to the corresponding uptake in NaCl buffer by one-way ANOVA with Dunnett’s test (**A**), by unpaired *t*-test (**B**,**D**), or by two-way ANOVA with Sidak’s multiple comparisons test (**C**).

To determine the driving force of MCT13, the uptake of cephradine was measured in several Hanks balanced salt solution (HBSS)-modified buffers (Figure 3A). MCT13-mediated cephradine uptake was significantly increased 3.8–6.2-fold in buffers in which NaCl was replaced with KCl or K-gluconate (KCl buffer or KGluconate buffer), compared with that in the HBSS buffer (NaCl buffer). In contrast, cephradine uptake was unchanged in other HBSS-modified buffers (mannitol buffer and NaGluconate buffer). Furthermore, we examined the effect of membrane potential on cephradine uptake by Ba^2+^ preincubation and FCCP co-incubation (Figure 3B). Cephradine uptake in the NaCl buffer was increased by pretreatment with Ba^2+^, which is a non-specific K^+^ channel blocker, and decreases membrane potential slowly [33]. Treatment with FCCP, which is a metabolic inhibitor that rapidly depolarizes plasma membrane potential [34], also increased cephradine uptake. MCT13-mediated cephradine uptake was enhanced by depletion of membrane potential, suggesting that the uptake process may be suppressed by an efflux driven by the membrane potential. Considering these results, the time-dependent uptake of cephradine by MCT13 was re-evaluated in KCl buffer (Figure 3C). Cephradine uptake in a KCl buffer was linearly increased for 5 min, and it was greater compared with that in mannitol buffer (Figure 2A and Figure 3C). Next, we evaluated the pH dependence of MCT13-mediated cephradine uptake in the KCl buffer (Figure 3D). The uptake activity was highest at pH 7.4 and decreased at pH 6.5 and pH 5.5. Kinetics analysis showed that cephradine uptake was saturable, and the K_m_ value was calculated as 2.83 ± 0.63 mM. These data imply that MCT13 transports cephradine as a substrate. In addition, we also confirmed that EGFP-tagged MCT13 transports cephradine as a substrate with a K_m_ value of 3.95 ± 0.53 mM, of which the uptake is enhanced by the membrane potential, the same as untagged MCT13 (Figure S2).

**Figure 4:**
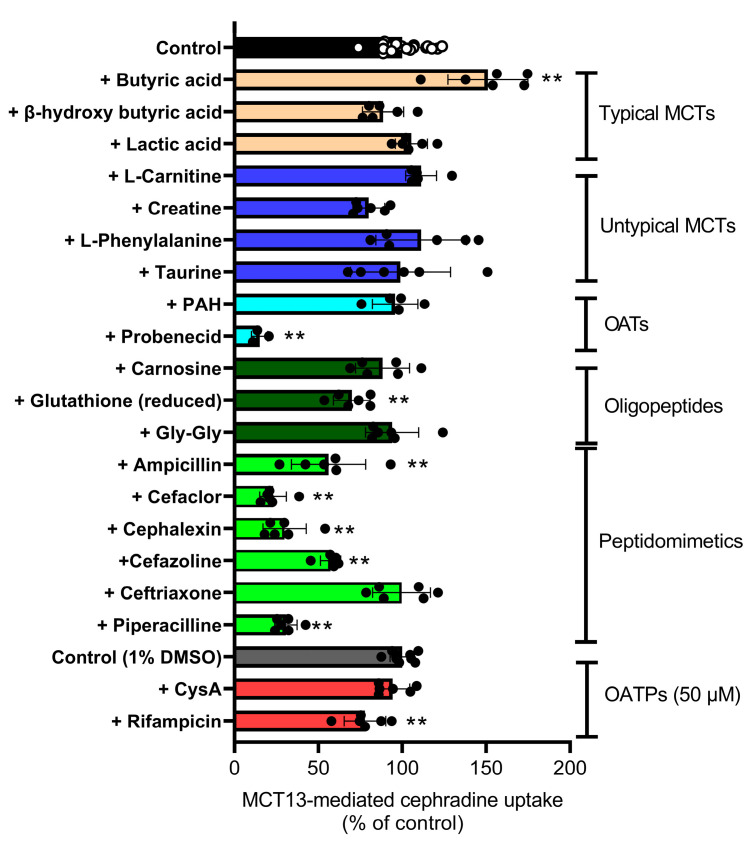
Inhibitory profiles of transporter-substrates/inhibitors on MCT13-mediated cephradine uptake. Uptake of cephradine was measured at 1000 μM in KCl buffer for 5 min, using HEK293T cells expressing MCT13 and/or CD147. The substrates/inhibitors of transporters were used at 10 mM except for cyclosporine A (CysA) and rifampicin at 50 μM. MCT13-mediated uptake was calculated by subtracting the uptake amount of mock-transfected cells from that of MCT13-transfected cells. Each bar represents the mean ± S.D. (*n* = 6), except for the control (*n* = 15), control (1% DMSO) (*n* = 9), and probenecid (*n* = 3). The small sample number in the case of probenecid was due to several measurements that were below the detection limit and did not appear in the graph. CysA and rifampicin were used in KCl buffer containing DMSO at a final concentration of 1%(*v*/*v*). ** *p* < 0.01, compared with the corresponding control by one-way ANOVA with Dunnett’s multiple comparisons test.

The inhibitory effects of substrates and inhibitors of known transporters on MCT13-mediated cephradine uptake were examined (Figure 4). MCT13-mediated cephradine uptake was not inhibited by SCFAs (e.g., lactic acid, butyric acid, and β-hydroxybutyric acid), L-carnitine, creatine, and amino acids, which are substrates/inhibitors of typical and atypical MCTs. In contrast, cephradine uptake was significantly decreased to its maximum value of 23% by cephalexin, ampicillin, cefaclor, and piperacillin, which are PEPT substrates/inhibitors. Cephazolin and glutathione partially inhibited uptake by 43% and 30%, respectively, although these were not PEPT substrates/inhibitors. Probenecid and rifampicin, which are typical inhibitors of organic anion transporters (OATs) and/or organic anion transporter polypeptides (OATPs), inhibited uptake; however, other OAT and/or OATP inhibitors (*p*-aminohippuric acid (PAH) and cyclosporine A (CysA)) did not have an effect. However, it is possible that the inhibitor concentration of OATPs may not be high enough to substantially inhibit the uptake of cephradine at 500 μM. The inhibitory profile of MCT13 appears similar to that of PEPTs rather than MCTs, OATs, and OATPs under these uptake conditions.

### 3.4. Enhancement of the Directional Transport of Cephradine across Caco-2 Cells by MCT13

**Figure 5:**
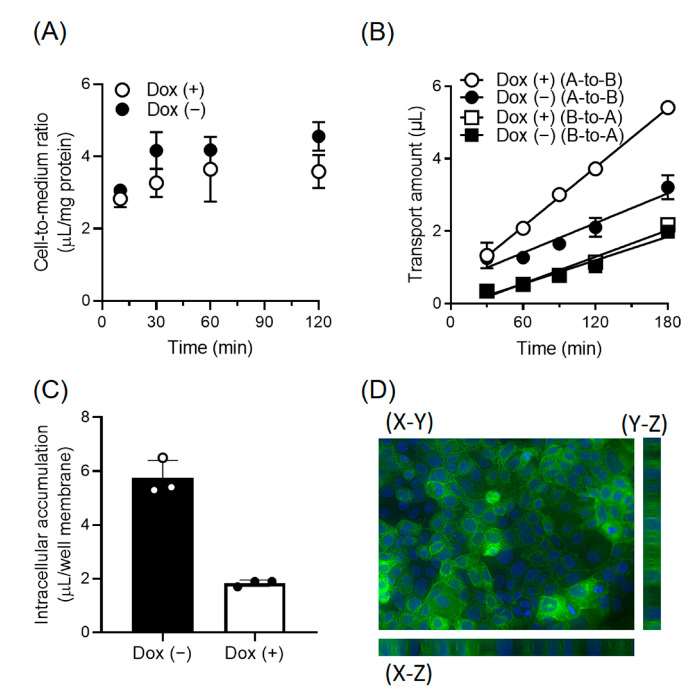
MCT13 enhances cephradine permeation across Caco-2 cells. MCT13 was induced in Caco-2-Tet-MCT13 cells by culturing in the medium including doxycycline at 5 μg/mL and sodium butyrate at 5 mM (Dox [+]) or sodium butyrate at 5 mM (Dox [−]) for 48 h. (**A**) Caco-2-Tet-MCT13 cells were seeded on 24-well plates. Uptake of cephradine was measured by the cells using HBSS buffer (pH 6.0). (**B**) Directional transport of cephradine across Caco-2-Tet-MCT13 cells. Caco-2-Tet-MCT13 cells were cultured on a Falcon cell culture insert membrane for 24–27 days and treated with doxycycline and sodium butyrate. Transport of cephradine (500 μM) from apical-to-basal chamber ((**A**)-to-(**B**)) or basal-to-apical chamber ((**B**)-to-(**A**)) was measured. HBSS buffer (pH 6.0 and pH 7.4) was used as a transport buffer for apical and basal chambers, respectively. (**C**) Intracellular accumulation of cephradine in Caco-2-Tet-MCT13 cells. Cephradine accumulation in Caco-2-Tet-MCT13 cells was evaluated after 3 h of A-to-B directional transport study. (**D**) Localization analysis of induced MCT13 in Caco-2 cells. Caco-2-Tet-MCT13 cells were cultured on a Falcon cell culture insert membrane. The nuclei were stained by DAPI. MCT13 (green) and nuclei (blue) were observed by confocal fluorescence microscopy. Each point represents the mean ± S.D. (*n* = 3). *p* < 0.01, compared to the corresponding Caco-2-Tet-MCT13 cells treated with only sodium butyrate by two-way ANOVA with Sidak’s multiple comparisons test (**B**) or unpaired *t*-test (**C**).

To determine the localization and function of MCT13 in polarized intestinal epithelial cells, we prepared Caco-2 cells, in which EGFP-tagged MCT13 expression was driven by conditional Tet-on promoters (Caco-2-Tet-MCT13). MCT13 expression was induced by doxycycline and sodium butyrate, which enhances doxycycline induction [19]. We examined the uptake of cephradine by Caco-2-Tet-MCT13 cells cultured on plastic dishes (Figure 5A). There was no significant difference in cephradine uptake between MCT13-induced and uninduced cells. Next, the directional transport of cephradine across Caco-2-Tet-MCT13 cells cultured on cell culture inserts was evaluated (Figure 5B). Apical-to-basal chamber (A-to-B) transport was significantly increased by MCT13 induction, although basal-to-apical chamber (B-to-A) transport was unchanged. The intracellular accumulation of cephradine in MCT13-induced cells was significantly decreased after 3 h in A-to-B transport experiments (Figure 5C). In addition, the permeability of lucifer yellow, a paracellular permeability marker, remained unchanged in both directions (Figure S4). The induced MCT13 was primarily localized at the basolateral membrane of Caco-2-Tet-MCT13 cells (Figure 5D). These results suggest that MCT13 functions as a releaser of cephradine at the basolateral membrane of Caco-2 cells.

### 3.5. Regulation of Intracellular Oligopeptide Levels by MCT13

**Figure 6:**
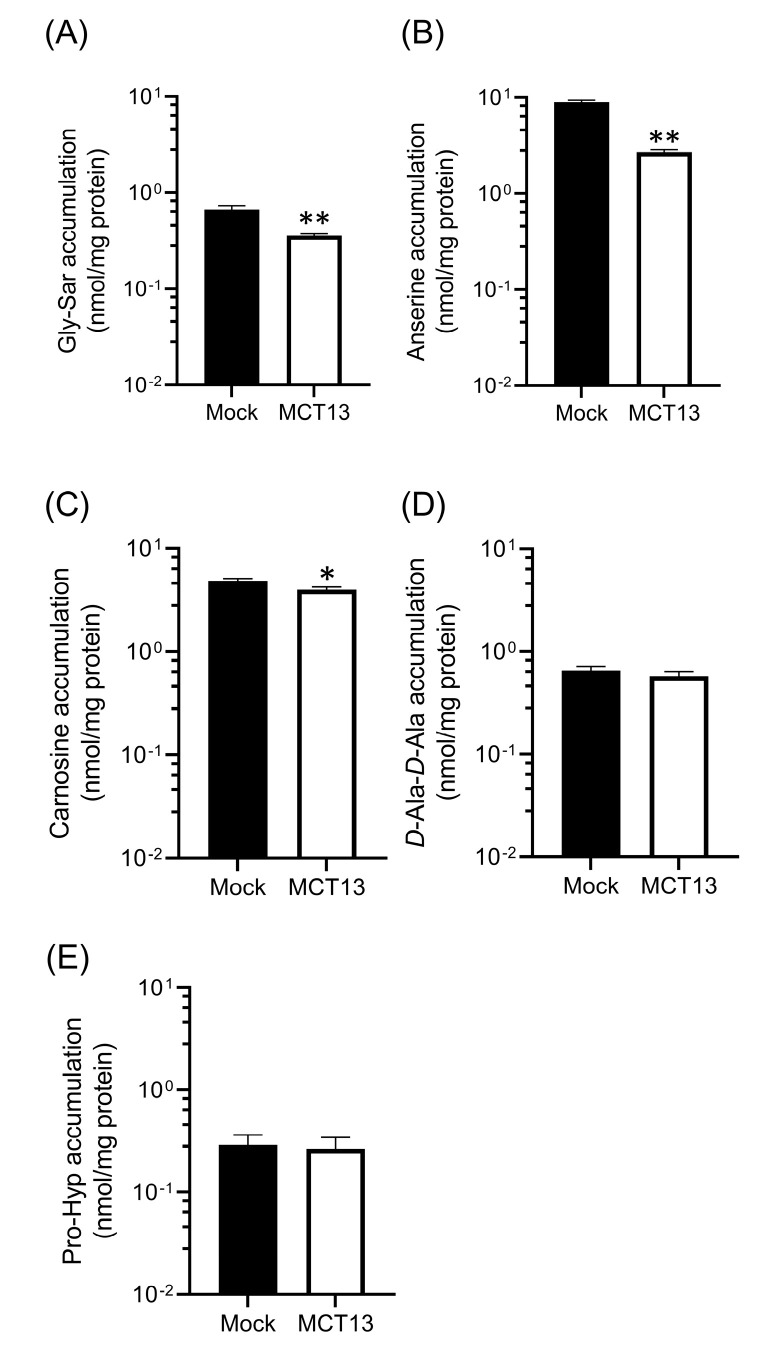
MCT13 suppresses intracellular accumulation of oligopeptides. HEK293T cells expressing MCT13 and CD147 were cultured with or without (**A**) Gly-Sar, (**B**) anserine, (**C**) carnosine, (**D**) *D*-Ala-*D*-Ala, or (**E**) Pro-Hyp at 500 μM for 24 h. Each bar represents the mean ± S.D. (*n* = 3). *, *p* < 0.05, **, *p* < 0.01, compared to the corresponding accumulation in mock-transfected cells by unpaired *t*-test.

We examined the effect of MCT13 on the intracellular accumulation of oligopeptides because MCT13 is expressed not only in the intestinal epithelia, but also in various cells, such as hepatocytes. We compared the intracellular levels at a steady state in MCT13-transfected and mock-transfected cells co-cultured with oligopeptides exhibiting peptidase resistance (Figure 6). The intracellular levels of Gly-Sar, carnosine, and anserine were significantly decreased in MCT13-transfected cells, although that of *D*-Ala-*D*-Ala and prolylhydroxyproline (Pro-Hyp) were unchanged. These data suggest that MCT13 suppresses the intracellular accumulation of several bioactive oligopeptides not only in the intestine but in other tissues.

## 4. Discussion

PEPT1 contributes to the transport of oligopeptides and peptidomimetics through the brush-border (apical) membrane of intestinal epithelia; however, little is known about basolateral membrane transporters, which are important for their efficient directional transport. In the present study, we demonstrated that MCT13, a transporter associated with type 2 diabetes, mediates the transport of cephradine, a probe for peptide transport systems, and oligopeptides such as Gly-Sar, anserine, and carnosine. We also found that MCT13 can function as a releaser of cephradine at the basolateral membrane of polarized intestinal epithelial cells. Our results provide insight into the physiological roles of MCT13 and the molecular mechanisms of peptide transport in intestinal epithelial cells.

We showed that MCT13 may be a novel transporter involved in the permeation of oligopeptides and peptidomimetics at the basolateral membrane of intestinal epithelia (Figure 5). Inui et al. reported that a specific basolateral transporter is involved in the efflux of cephradine from Caco-2 cells [35]. Cephradine uptake from the basolateral membrane of Caco-2 cells was slightly enhanced under acidic conditions [36]; however, our results indicated that MCT13-mediated cephradine uptake was decreased under acidic conditions (Figure 3C). The pH-dependent profile did not completely correspond to that previously reported for basolateral oligopeptide transporters in Caco-2 cells. MCT13 may be another molecule of a basolateral oligopeptide transporter that is different from previously reported transporters, although it is expressed in the human duodenum, jejunum, and Caco-2 cells (Figure S3). However, there have been conflicting reports regarding the H^+^-dependence of oligopeptide transport by using Gly-Sar as a transport probe at the basolateral membrane. Additional research is required to determine the molecules that are responsible for the basolateral oligopeptide transport in Caco-2 cells.

A variant of *SLC16A13* gene was reported to be related to the development of diabetes mellitus in the Chinese population [27]. Our study revealed that human MCT13 mediates the transport of oligopeptides, raising the possibility that it may be associated with type 2 diabetes by regulating bioactive oligopeptide transport. Carnosine and anserine are present in the skeletal muscles of vertebrates, such as chicken and beef, and are ingested from the diet or synthesized in the body. Supplementation with carnosine and anserine decreases blood glucose and affects lipid profiles in human and animal studies [7,37]. If the activity of MCT13 is altered by the variant of the *SLC16A13* gene, the plasma concentration of bioactive oligopeptides such as anserine and carnosine might be affected by changing the absorption from gastrointestinal tracts. There are various types of oligopeptides present in the diet and resulting from protein degradation in the body. Thus, it is challenging to identify endogenous substrates of oligopeptides that are associated with type 2 diabetes. On the other hand, MCT13-deficient mice had reduced intrahepatocellular lactate levels and increased AMP-activated protein kinase (AMPK) activation, which results in reduced hepatic lipid accumulation and increased insulin sensitivity when fed with a high-fat diet [28]. Dietary conditions are likely to be related with the pathophysiological phenotype of MCT13, although it is arguable that the phenotype of mice is relevant to the variance in the human gene.

We identified the peptidomimetic cephradine as a novel substrate for MCT13 (Figure 3E), which enabled us to further characterize the transport function of MCT13. Cephradine is an anionic peptide-like compound and is stable under biological conditions. Thus, it was used as a probe to measure peptide absorption in animal intestines [38]. We found that the MCT13-mediated uptake of cephradine was increased under conditions of membrane-potential depletion using KCl buffer and FCCP (Figure 3A,B). Moreover, we showed that MCT13 decreased the accumulation of several bioactive peptides in MCT13-transfected cells under normal culture conditions (Figure 6) and mediated the efflux of cephradine from the intracellular space of Caco-2 cells to the basolateral side (Figure 5). These results suggest that MCT13 may have the ability to mediate the uptake and efflux of cephradine across the plasma membrane by two distinct modes. One is a mode for the uptake of cephradine and related peptidomimetics, which might be a facilitative process without requiring an ion gradient. The other mode is for the efflux of cephradine but also of several dipeptides like anserine and carnosine, which might be dependent on membrane potential determined by ion gradients. These modes are supported by the distinct dipeptide efflux activity (Figure 2E and Figure 6B,C), compared with the faint uptake activity (Figure 2C,D). Furthermore, the results of the inhibition study using the uptake method indicated that cephradine uptake was more responsive to peptidomimetics such as β-lactam antibiotics compared to dipeptides and tripeptides (Figure 4 and Figure S5). It seems that the substrate affinity or substrate recognition of MCT13 may be different between the two modes. It is difficult to examine the effect of ion gradients on MCT13-mediated cephradine efflux because it is challenging to load an equal amount of cephradine into both MCT13-transfected and mock-transfected cells. Further investigation about the transport modes is needed.

We found that MCT13 is an oligopeptide transporter with even lower affinities compared with PEPT1. Our results show that MCT13-mediated cephradine uptake was not inhibited by endogenous dipeptides and tripeptides at 10 mM (e.g., glycylglycine (Gly-Gly), carnosine, and glutathione), but inhibited at a 50 mM concentration (Figure 4 and Figure S4). The intestinal absorption of di- and tripeptides from the lumen into epithelial cells is primarily mediated by PEPT1, which is a low-affinity/high-capacity peptide transporter that exhibits a wide range of affinity constants (2 μM to 15 mM) for several endogenous dipeptides and β-lactam antibiotics [39,40,41,42]. Considering that the substrates of PEPT1 are concentrated by a proton gradient into the intestinal epithelial cells of the gastrointestinal tract, the lower affinity of MCT13 for endogenous oligopeptides may be suitable for the efficient release into the blood. Further studies of the structures and chemical properties of compounds are necessary to obtain a complete understanding of the substrate specificity of MCT13.

MCT13 was shown to interact with CD147 and GP70 in a yeast hybrid system [43]; however, it was unclear whether this interaction affects the localization and function of MCT13. Our results indicated that interactions between MCT13 with CD147 enhance plasma membrane localization in HEK293T cells (Figure 1). We also confirmed that the interactions increased the transport activity of MCT13 (Figure S6). CD147 appears to be localized at the blood side (basolateral) of the membrane in small intestinal tissues based on the human protein atlas database [24]; thus, the interaction of MCT13 with CD147 may affect transport function in the intestinal epithelia. However, further studies are needed to determine whether CD147 is an interaction partner with MCT13 in the intestinal epithelial cells.

Notably, mouse Mct13 was shown to transport lactic acid as a substrate with a K_m_ value of 0.47 μM [28]; however, our results indicated that cephradine uptake by human MCT13 was not inhibited by lactic acid at 10 mM. In addition, other SCFAs (e.g., butyric acid and β-hydroxybutyric acid) did not inhibit uptake. Based on these results, human MCT13 is either unlikely to transport lactate as a high-affinity substrate, or it may transport lactate with the use of a different binding pocket than the one used for cephradine and related peptidomimetics/oligopeptides.

In the present study, we examined the transport characteristics of MCT13 by uptake experiments, although MCT13 is also likely to function as an efflux transporter under physiological conditions. The data obtained may not completely correspond to the physiological efflux transport characteristics of MCT13; however, the uptake method using KCl buffer is convenient and applicable for the identification of MCT13 substrates. Dawson et al. found that the membrane potential-dependent bile acid efflux transporter (OSTα/OSTβ)-mediated uptake was stimulated by the same method using a KCl buffer [44]. In fact, we successfully identified oligopeptides and peptidomimetics as substrates for MCT13.

## 5. Conclusions

In summary, we found that human MCT13 functions as a novel transporter for oligopeptides and peptidomimetics. Cephradine was identified as a probe substrate for MCT13, and transport is dependent upon membrane potential. In polarized intestinal epithelia, MCT13 was primarily localized at the basolateral membrane and functions as a releaser of substrates. Furthermore, plasma membrane expression of MCT13 was enhanced through its interaction with ancillary protein CD147 under in vitro conditions. These findings provide further insight into the pathophysiological role of MCT13, which is associated with type 2 diabetes.

## Data Availability

All data are accessible in the manuscript or supporting information.

## Supplementary Materials

The following supporting information can be downloaded at: https://www.mdpi.com/article/10.3390/nu15163527/s1, Figure S1: Effect of ancillary proteins on localization of MCT13; Figure S2: Characteristics of cephradine uptake by EGFP-tagged MCT13; Figure S3: mRNA expression of MCT13 in human duodenum, jejunum, and intestinal model cell lines; Figure S4: Permeation of lucifer yellow across Caco-2-Tet-hMCT13 cells; Figure S5: Inhibitory effect of Gly-Gly and carnosine on MCT13-mediated cephradine uptake; Figure S6: Effect of CD147 and GP70 on MCT13-mediated cephradine uptake; Table S1: LC-MS/MS measurement condition.
