# Supplementary material for: Monocarboxylate Transporter 13 (MCT13/SLC16A13) Functions as a Novel Plasma Membrane Oligopeptide Transporter
Source: Nutrients. 2023 Aug 10;15(16):3527. doi: 10.3390/nu15163527 (PMC10458055; doi:10.3390/nu15163527)
Supplement: Supplementary file 1 [file nutrients-15-03527-s001.zip › nutrients-2520962-supplementary.pdf]

SUPPORTING INFORMATION

Monocarboxylate Transporter 13 (MCT13/SLC16A13) Functions as a Novel Plasma Membrane Oligopeptide Transporter

Kei Higuchi [1], Misato Kunieda [1], Koki Sugiyama [1], Ryuto Tomabechi [1,2], Hisanao Kishimoto [1] and Katsuhisa Inoue [1,*]

1 Department of Biopharmaceutics, School of Pharmacy, Tokyo University of Pharmacy and Life Sciences, 1432-1 Horinouchi, Tokyo 192-0392, Japan; higuchi@toyaku.ac.jp (K.H.); y174054@toyaku.ac.jp (M.K.); y141106@toyaku.ac.jp (K.S.); tomabechi.ryuto@kitasato-u.ac.jp (R.T.); kisimoto@toyaku.ac.jp (H.K.)

2 Laboratory of Pharmaceutics, Kitasato University School of Pharmacy, 5-9-1 Shirokane, Tokyo 108-8641, Japan

* Correspondence: kinoue@toyaku.ac.jp; Tel.: +81-42-676-3126

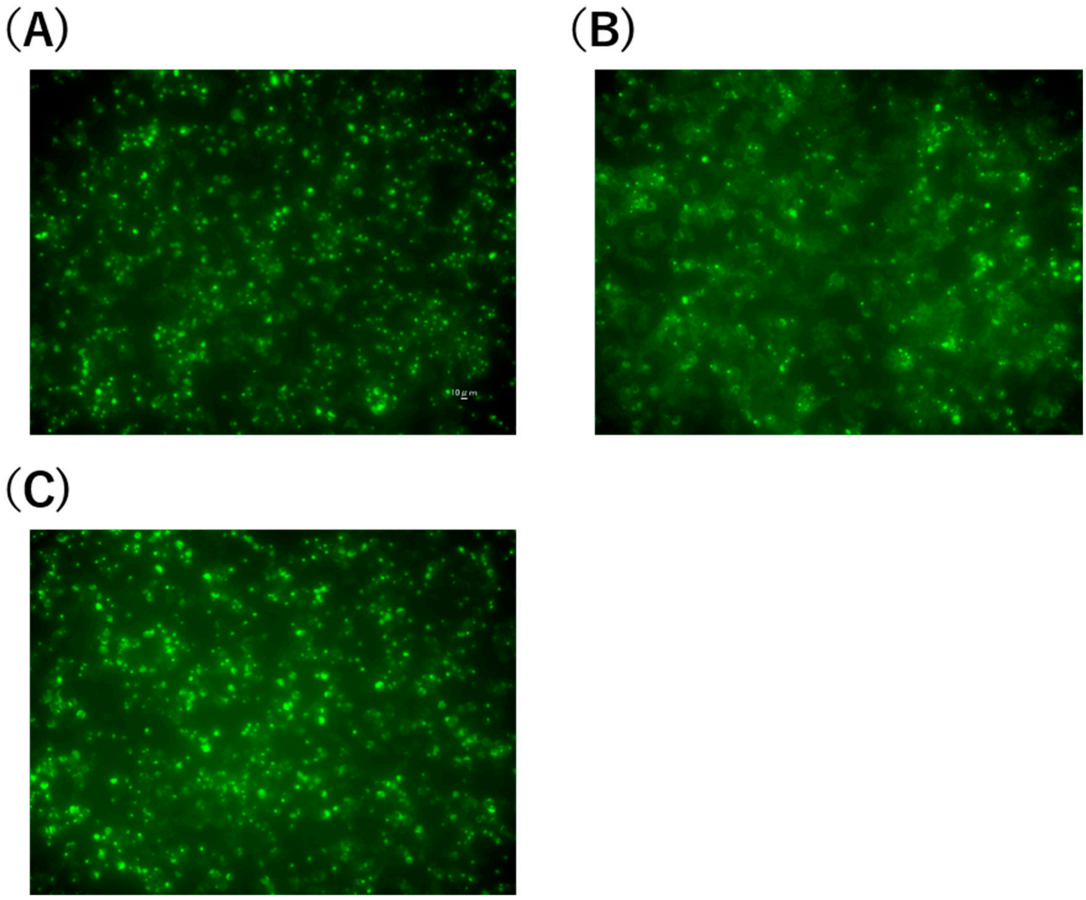

Figure S1 Effect of ancillary proteins on localization of MCT13

HEK293T cells were transfected with plasmids of EGFP-tagged MCT13 and empty vector **(A)**, CD147 **(B)**, or GP70 **(C)**. The EGFP-signal were observed by fluorescence microscopy at low magnification.

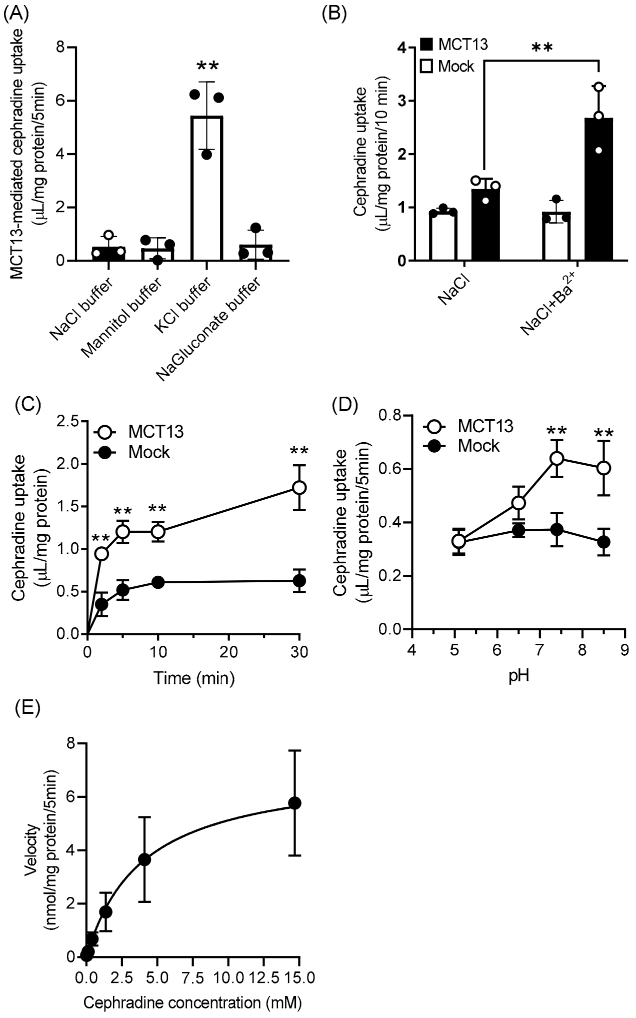

Figure S2 Characteristics of cephradine uptake by EGFP-tagged MCT13

Cephradine uptake was measured at 500 μM in HEK293T cells expressing EGFP-tagged MCT13 and CD147. **(A)** Effects of extracellular ions on EGFP-tagged MCT13-mediated cephradine uptake. Uptake in HBSS buffer (pH 7.4) or HBSS-modified buffer (pH 7.4) was measured. **(B)** Effect of Ba^{2+} on EGFP-tagged MCT13-mediated cephradine uptake. The cells were preincubated with NaCl buffer with or without 2 mM Ba^{2+}. Cephradine uptake in preincubated cells was measured in the same buffer (pH 7.4). **(C)** Cephradine uptake was measured in KCl buffer (pH 7.4) for designed time. **(D)** Effect of extracellular pH on EGFP-tagged MCT13-mediated cephradine uptake. The uptake was measured in Mannitol buffer at 5.5 6.5, 7.4, and 8.5. **(E)** Concentration dependence of EGFP-tagged MCT13-mediated cephradine uptake. The uptake at 0.1–15 mM was measured. Each point represents the mean ± S.D. (n = 3). **, $p < 0.01$, compared to the corresponding uptake in NaCl buffer by one-way ANOVA with Dunnett's test (A), by unpaired t-test (B, D), or by two-way ANOVA with Sidak's multiple comparisons test (C).

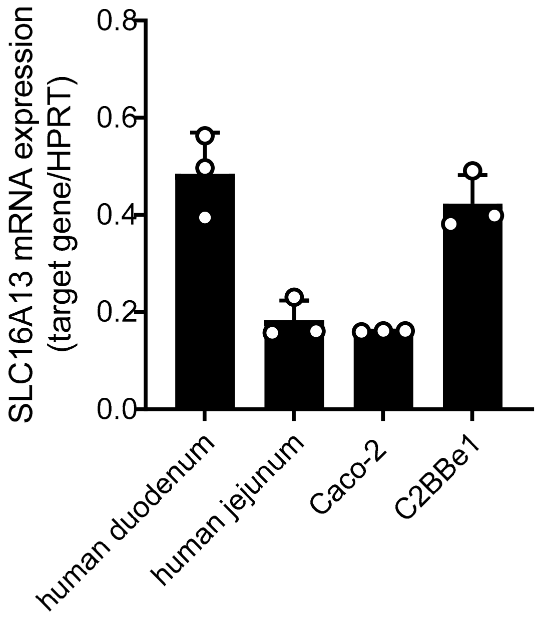

Figure S3 mRNA expression of MCT13 in human duodenum, jejunum, and intestinal model cell lines

Quantitative-RT-PCR was performed with Thunderbird SYBR qPCR Mix (Toyobo) using 1.3 ng of sample cDNA. Primer sequences were as follows: SLC16A13 forward, 5’-CAGTTTGGGAGCCCGGTA-3’; SLC16A13 reverse, 5’-GGTGGGTCAAGGAAG TAGCA-3’; HPRT forward, 5’-GCGTCGTGATTAGCGATGATGAAC-3’; HPRT reverse, 5’-CCTCCCATCTCCTTCATGACATCT-3’. Amplification and detection for Quantitative-PCR were carried out on an AriaMx Real-Time PCR instrument (Agilent Technologies, Inc.), and the results were analyzed by AriaMx Software version 1.71. The relative mRNA expression was determined by the $2^{-\Delta\Delta}$ Ct method. HPRT was used as a housekeeping gene to normalize the relative expression level in samples.

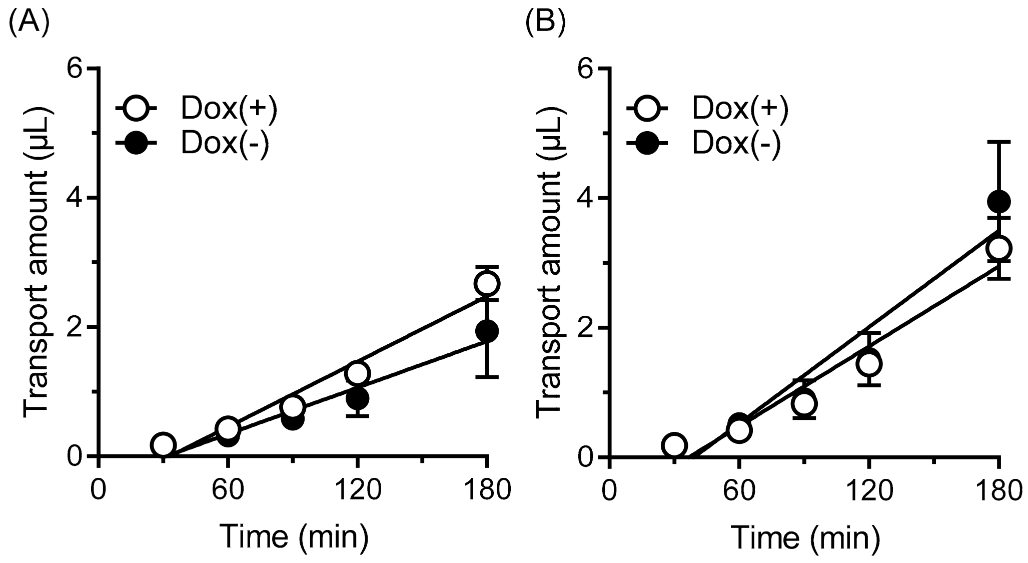

Figure S4 Permeation of lucifer yellow across Caco-2-Tet-hMCT13 cells.

Caco-2-Tet-MCT13 cells were cultured on a Falcon cell culture insert membrane. MCT13 was induced by culturing in the medium including doxycycline at 5 μg/mL and sodium butyrate at 5 mM (Dox [+]), or sodium butyrate at 5 mM (Dox [−]), for 48 hr. Transport of lucifer yellow (500 μM) from apical-to-basal chamber (A-to-B) or basal-to-apical chamber (B-to-A) were measured. HBSS buffer (pH 6.0 and pH 7.4) was used as transport buffer for apical and basal chamber, respectively.

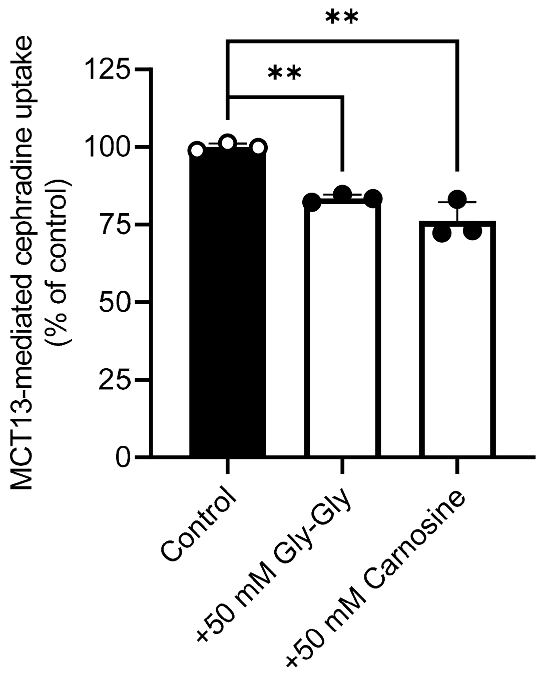

Figure S5 Inhibitory effect of Gly-Gly and carnosine on MCT13-mediated cephradine uptake.

HEK293T cells were transfected with hMCT13/pcDNA3.1/Hygro or empty-vector with CD147/pCI-Neo. Uptake of cephradine by the cells was measured in KCl buffer for 5 min. The substrates/inhibitors of transporters were used at 50 mM. MCT13-mediated uptake was calculated by subtracting the uptake amount of mock-transfected cells from that of MCT13-transfected cells. Each bar represents the mean ± S.D. (n = 3). ** $p < 0.01$, compared with the corresponding control by one-way ANOVA with Dunnett's multiple comparisons test.

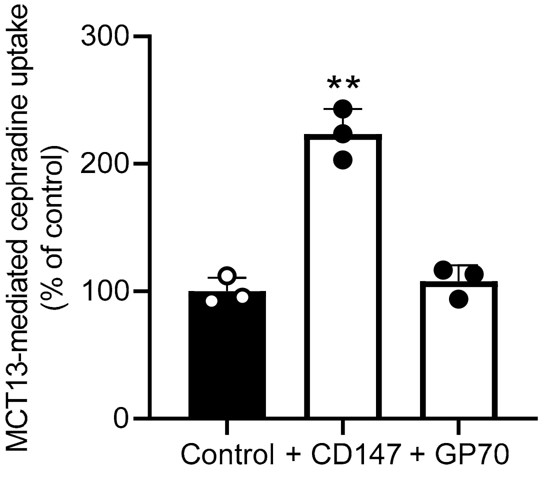

Figure S6 Effect of CD147 and GP70 on MCT13-mediated cephradine uptake. HEK293T cells were transfected with MCT13 and/or CD147 or GP70. Uptake of cephradine by those cells was measured in KCl buffer (pH 7.4) for 5 min. Each bar represents the mean ± S.D. (n = 3). **, $p < 0.01$, compared to the corresponding mock-transfected cells (control) by one-way ANOVA with Dunnett's test.

Table S1 LC-MS/MS measurement condition

**LC method**	**Gly-Sar, cephradine, carnosine, anserine, Pro-Hyp, D-Ala-D-Ala**	
Column	Intrada Amino acid 50 × 3 mm I.D., 3 μm	
Column temperature	35°C	
Mobile Phase	solvent A (ammonium formate buffer [20 mM])	
	solvent B (acetonitrile)	
Gradient	1) Gly-Sar, cephradine, Pro-Hyp, D-Ala-D-Ala	
	10:90 (A:B) (0-1.0 min)	
	10:90 (A:B) to 100:0(A:B) (1.0-2.2 min)	
	100:0 (A:B) (2.2-3.0 min)	
	100:0 (A:B) to 10:90(A:B) (3.0-3.1 min)	
	10:90 (A:B) (3.1-3.8 min)	
	2) Carnosine, anserine	
	10:90 (A:B) (0-1.0 min)	
	10:90 (A:B) to 100:0(A:B) (1.0-2.2 min)	
	100:0 (A:B) (2.2-3.0 min)	
	100:0 (A:B) to 10:90(A:B) (3.0-3.1 min)	
	10:90 (A:B) (3.1-3.8 min)	
Flow rate	0.6 mL/min	
Injection volume	2.5 μL/sample	
**MS/SM conditions**		
Ionization mode	ESI (+)	
Desolvation temperature	500°C	
Ion source temperature	150°C	
Spray voltage	3.5 kv	
Collision energy	10V (Gly-Sar)	10V (cephradine)
	14V (carnosine)	22V (anserine)
	20V (Pro-Hyp)	12V (D-Ala-D-Ala)
Q1/Q3 (m/z)	147/90(Gly-Sar)	50/158(cephradine)
	227/110(carnosine)	241/109(anserine)
	228/70 (Pro-Hyp)	161/90 (D-Ala-D-Ala)
